# Olfaction and Quality of Life in Patients with Eosinophilic CRS Undergoing Endoscopic Sinus Surgery

**DOI:** 10.1055/s-0043-1772494

**Published:** 2024-02-16

**Authors:** Miguel Soares Tepedino, Richard Louis Voegels, Rogério Pezato, Andrew Thamboo, Eduardo Macoto Kosug, Ana Clara Miotello Ferrão, Raíssa de Figueiredo Neves, Valéria Maria Barcia Castilla, Luis Carlos Gregório

**Affiliations:** 1 Department of Otolaryngology, Universidade do Estado do Rio de Janeiro, Rio de Janeiro, RJ, Brazil; 2 Department of Otolaryngology, Policlinica de Botafogo, Rio de Janeiro, RJ, Brazil; 3 Department of Otorhinolaryngology and Head and Neck Surgery, Universidade Federal de São Paulo, São Paulo, SP, Brazil; 4 Department of Otolaryngology, University of São Paulo, São Paulo, SP, Brazil; 5 Department of Otolaryngology and Head & Neck Surgery, ENT Research Laboratory, Universidade Federal de São Paulo, São Paulo, SP, Brazil; 6 Division of Rhinology, University of British Columbia, Vancouver, Canada; 7 Department of ENT, Policlinica de Botafogo, Rio de Janeiro, RJ, Brazil

**Keywords:** Chronic rhinosinusitis, olfaction, surgery, quality of life

## Abstract

**Introduction** Chronic rhinosinusitis (CRS) is a common inflammatory disease. This high prevalence leads to high direct and indirect public health costs, which include medical visits, laboratory tests and imaging, pharmacotherapy, hospitalizations, and surgical treatment. Furthermore, CRS has a substantial impact on patient quality of life, affecting productivity and being a common cause of absence from work CRS-associated olfactory dysfunction is highly prevalent, the actual effectiveness of surgical intervention remains inconsistent. Although there are studies evaluating the postoperative course of patients with eosinophilic Chronic rhinosinusitis (eCRS) treated with high-volume budesonide irrigation, there is little objective information regarding the impact of this intervention on olfactory status and quality of life.

**Objective** To conduct a pre- and postoperative analysis of olfaction and quality of life in patients with eCRS treated with surgical intervention followed by high-volume budesonide nasal irrigation.

**Methods** Prospective, descriptive, uncontrolled study of patients with eCRS. All patients underwent pre- and postoperative nasal endoscopy, SNOT-22 questionnaire, and the University of Pennsylvania Smell Identification Text (UPSIT), always by the same previously trained examiner. The SNOT-22 questionnaire and the UPSIT were readministered to all patients at 3 months, 6 months, and 1 year postoperatively, and scores compared with those obtained preoperatively.

**Results** Twenty patients were included in the study, 13 males and 7 females, between the ages of 23 and 65; 8 patients had comorbid asthma. Quantitative evaluation using the UPSIT test showed a significant improvement in olfaction 3 months after surgery, which remained 6 months and 1 year after surgery (p = 0.0063).

There was no significant association between eosinophil concentrations in polypoid tissue and postoperative SNOT-22 and UPSIT results. Patients with tissue eosinophils >50 had a lower preoperative UPSIT score. As early as 3 months postoperatively, a significant improvement in quality of life was already noticeable, as represented by a decrease in SNOT-22 values, which persisted through the 1-year postoperative follow-up evaluation (p = 0.0005). Quantitative evaluation using the UPSIT test showed a significant improvement in olfaction 3 months after surgery, which remained 6 months and 1 year after surgery (p = 0.0063).

**Conclusion** Surgery effectively controlled eCRS in patients who adhered to high-volume budesonide nasal irrigation postoperatively. There were significant improvements in quality of life and olfaction, which persisted at least up to one year postoperatively.

## Introduction

Chronic rhinosinusitis (CRS) is a common inflammatory disease, with an estimated worldwide prevalence of 5 to 15%. 1 2 3 4 In the United States, this high prevalence leads to high direct and indirect public health costs, which include medical visits, laboratory tests and imaging, pharmacotherapy, hospitalizations, and surgical treatment. Furthermore, CRS has a substantial impact on patient quality of life, affecting productivity and being a common cause of absence from work. 5 6 7

EPOS divides CRS into two main subtypes, primary or secondary and localized or diffuse. Primary diffuse CRS is divided into eosinophilic or type 2 (eCRS) and non-eosinophilic or non-type 2 (neCRS) phenotypes, as determined by histological quantification of eosinophilic cells (10 eosinophils per high-powered field at 400x magnification). Patients with eCRS often have difficult-to-control disease 8 compered to neCRS patients, making of interest to evaluate the impact of eosinoplil concentrations on olfactory response after treatment in CRS with nasal polyps' patients.

The initial treatment of CRS is usually clinical, followed, if necessary, by surgical intervention, with adjunctive medication in the postoperative period. The clinical effects of corticosteroids are achieved by a combination of anti-inflammatory mechanisms involving suppression of pro-inflammatory genes and increased transcription of anti-inflammatory genes, with consequent reductions in the migration of inflammatory cells, chemotactic factors, and cell adhesion molecules. 9

Although systemic corticosteroids exert greater control over the inflammatory pathways implicated CRS, they carry many significant side effects, such as agitation, dyspepsia, increased intraocular pressure, exacerbation of pre-existing hypertension, weight gain, fluid retention, osteoporosis, avascular necrosis of the femoral head, and hypothalamic-pituitary-adrenal axis dysfunction. 10 Therefore, there is an ongoing effort to replace systemic therapy with topical nasal therapy to achieve control of CRS. 11 Direct administration of the drug to the affected tissues allows for a higher local concentration with less systemic absorption, increasing the effectiveness of treatment and reducing side effects. 12

CRS-associated olfactory dysfunction is highly prevalent, ranging from 48% to 83%—depending on how olfactory dysfunction is defined—in studies. It is well established that the surgical approach can improve olfactory function in these patients, since it resects the inflamed tissue, increasing airflow and improving the inflammatory response. 13 Nevertheless, the actual effectiveness of surgical intervention remains inconsistent. 14

Wide opening of the paranasal sinuses has been considered a fundamental step in the treatment of difficult-to-control CRS, as it establishes a single sinonasal cavity that can be irrigated with topical agents. 15 However, there is a perception that the corticosteroid nasal sprays available on the market do not ensure adequate delivery of the medication to the paranasal sinuses. The off-label postoperative use of budesonide solution delivered via a high-volume, low-pressure system allows the medication to reach the paranasal cavities and remain in place for a prolonged period, improving disease control and prognosis in CRS. 16

Although there are studies evaluating the postoperative course of patients with eCRS treated with high-volume budesonide irrigation, there is little objective information regarding the impact of this intervention on olfactory status and quality of life.

In this study we aim to conduct a pre- and postoperative analysis of olfaction and quality of life in patients with eCRS treated with surgical intervention followed by high-volume budesonide nasal irrigation.

## Materials and Methods

Adult patients aged 18 to 65 years with a diagnosis of eCRS according to the EPOS2020 criteria were included in the study. All patient care and surgical interventions were performed at the same hospital, located in Rio de Janeiro, Brazil, from February 2018 through December 2020.

All participants provided written informed consent. The study was approved by the local Research Ethics Committee with opinion number43925121.7.0000.5282.

The following exclusion criteria were applied: age <18 years; history of sinonasal surgery; pregnancy; history or diagnosis of sinonasal malignancy; olfactory dysfunction associated to trauma and post-infectious; neurological diseases; immunosuppression and/or immunodeficiency; history of allergic reaction to corticosteroids; medical contraindications to topical corticosteroid therapy; suspected or confirmed glaucoma; uncompensated hypertension or diabetes with clinical contraindications to corticosteroid therapy; and use of topical and/or systemic antibiotics, antihistamines, or corticosteroids in the 30 days preceding surgery and postoperative evaluations.

### Study Design

Prospective, descriptive, uncontrolled study of patients with eCRS. All patients underwent pre- and postoperative nasal endoscopy. The SNOT-22 questionnaire and the University of Pennsylvania Smell Identification Text (UPSIT) were administered, always by the same previously trained examiner. The SNOT-22 questionnaire consists of 22 questions about possible symptoms associated with CRS. Each item is assigned a score from 0 to 5, where zero is absence of the symptom and five is the worst possible severity. Accordingly, higher total scores represent worse quality of life.

The UPSIT is scored on a scale of 0 to 40 (four cards, each with 10 different smells to be recognized), through which the respondent's olfactory function can be classified as normosmia, microsmia (mild, moderate, or severe), or anosmia.

The surgical approach was always performed by the same operator, who excised any polyps and opened all paranasal sinuses. The procedures were performed under endoscopic visualization, with a 4-mm, zero-degree and 45-degree Karl Storz scope, and zero-degree and 40-degree Medtronic shaver blades. In patients with polyps in the middle turbinate, a partial middle turbinectomy was performed. When the middle turbinates were preserved, they were sutured to the nasal septum with 4-0 Vicryl. Care was taken during the surgical procedure to preserve the mucosa and remove any loose bone fragments.

At the end of the procedure, Merocel®-type nasal packing was placed bilaterally to the roof of the ethmoid followed by a Doyle splint. Both were removed by the surgeon at 1-week follow-up. Patients were hospitalized for 24 hours and discharged on oral antibiotics (amoxicillin/clavulanate) and corticosteroids, in addition to nasal lavage with 0.9% saline solution. The corticosteroid of choice was prednisolone, 40 mg/day for 7 days. The budesonide used for topical therapy was always prepared by the same compounding pharmacy, at a concentration of 1 mg/4 mL. Patients were instructed to dilute one 4-mL vial in 500 mL of 0.9% saline solution and irrigate 60 mL of the solution into each nostril, using a 60-mL syringe, four times a day, for a total of 1 mg budesonide administered over 24 hours. Irrigation was started during the first (7-day) postoperative visit, after patient training, and patients were instructed to continue until the 1-year end-of-study visit.

Histological examination was performed by a pathologist through a Leica DM2000 binocular microscope at 400x magnification. The total number of eosinophils per high-power field (HPF) was counted in an average of 10 fields of view selected from most inflamed area of tissue 17

The SNOT-22 questionnaire and the UPSIT were readministered to all patients at 3 months, 6 months, and 1 year postoperatively, and scores compared with those obtained preoperatively.

### Statistical Analysis

The data generated were analyzed in STATA 14.2 (Stata Corp, Texas, USA). Results were assessed for normality of distribution by the Kolmogorov–Smirnov test. Spearman's test was used to test for correlations. Friedman's test was used to evaluated paired groups, and the Mann–Whitney *U* test was used to compare unpaired groups. P-values of less than 0.05 were considered significant.

## Results

Twenty patients were included in the study, 13 males and 7 females, between the ages of 23 and 65; 8 patients had comorbid asthma. Table 1 details the study findings.

**Table 1:** Study findings up to 1-year follow-up

Patient	Gender	Age	Lund -Kennedy preop	Asthma	Upsit preop	Upsit 3m	Upsit 6m	Upsit 1y	Snot preop	Snot 3m	Snot 6m	Snot 1y	Eosinophiles
**BM**	M	37	10	N	29	32	28	34	73	10	10	0	256/10 HPF
**CA**	M	64	8	N	6	17	15	20	38	6	13	11	123/10 HPF
**VS**	F	54	7	N	7	32	29	30	45	0	0	0	190/10 HPF
**RO**	M	53	8	N	15	32	34	36	65	9	13	9	170/10 HPF
**DL**	M	41	6	Y	20	30	34	33	45	20	12	16	32/10 HPF
**MF**	F	39	12	Y	6	35	27	28	83	4	5	7	163/10 HPF
**ES**	M	63	9	N	16	22	27	26	8	0	0	0	44/10 HPF
**LS**	F	52	10	Y	7	31	30	31	56	8	8	15	69/10 HPF
**JC**	M	58	6	Y	20	25	21	26	75	40	73	48	186/10 HPF
**MS**	F	38	10	Y	11	34	35	36	60	17	16	15	164/10 HPF
**MP**	M	41	8	N	14	17	23	23	41	8	8	8	47/10 HPF
**SS**	M	60	11	Y	13	14	14	15	49	13	13	7	239/10 HPF
**AC**	F	29	12	N	13	31	31	29	78	2	2	1	101/10 HPF
**LP**	M	40	10	N	19	17	15	15	52	8	7	7	67/10 HPF
**GA**	M	23	11	N	6	32	32	33	26	1	1	1	136/10 HPF
**AM**	F	25	8	N	16	25	27	34	56	16	13	11	47/10 HPF
**FC**	M	65	7	Y	4	17	27	30	46	15	5	1	254/10 HPF
**MC**	M	54	12	N	12	32	34	36	90	9	10	9	84/10 HPF
**DB**	F	26	6	N	30	31	34	35	73	8	8	7	31/10 HPF
**JO**	M	64	12	Y	7	23	35	35	65	5	4	4	126/10 HPF

Preop: preoperative. 3m: 3 months; 6m: 6 months; 1y: 1 year; HPF: high-powered field; N:no; Y: yes.

**Fig. 1:**
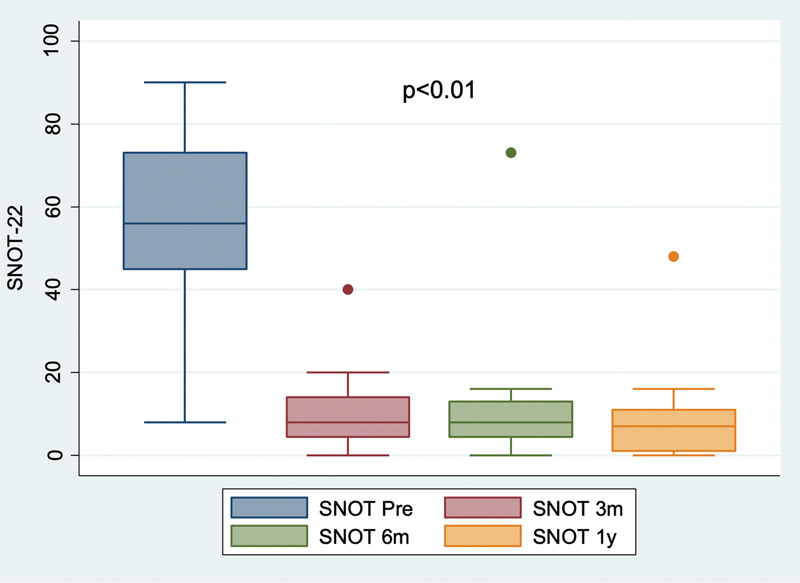
Progression of Sinonasal Outcome Test-22 (SNOT-22) score: preoperative and 3, 6, and 12 months postoperatively, Friedman's test was applied.

Patient quality of life was measured using the SNOT-22 questionnaire. As earlyas 3 months postoperatively, a significant improvement in quality of life was already noticeable, as represented by a decrease in SNOT-22 values, which persisted through the 1-year postoperative follow-up evaluation (p = 0.0005) ( Figure 1 ). No patient had normal preoperative SNOT-22 scores. At 1-year postoperative follow-up, however, 14 patients had scores <10, which are comparable to those of healthy individuals.

**Fig. 2:**
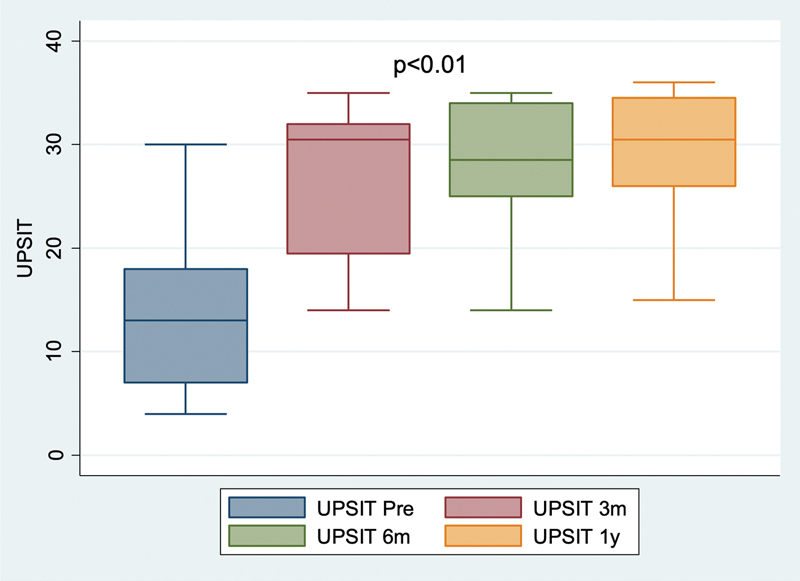
Progression of Unniversity of Pennsylvania Smell Identification Text (UPSIT) score: preoperative and 3, 6, and 12 months postoperatively, Friedman's test was applied.

Quantitative evaluation using the UPSIT test showed a significant improvement in olfaction 3 months after surgery, which remained 6 months and 1 year after surgery (p = 0.0063) ( Figure 2 ). Preoperatively, 2 patients were classified as having moderate microsmia, 3 with severe microsmia, and 15 with anosmia. One year after surgery, 6 patients were classified as having normosmia, 6 with mild microsmia, 4 with moderate microsmia, 2 with severe microsmia, and 2 with anosmia.

In 8 patients, the middle turbinate was resected bilaterally. There was no significant difference in postoperative course according to SNOT-22 and UPSIT scores. Asthma had no significant impact on SNOT-22 or UPSIT scores, before or after treatment.

Preoperative Lund-Kennedy scores ranged from 6 to 12, with a mean of approximately 9, demonstrating extensive nasal involvement.

**Fig. 3:**
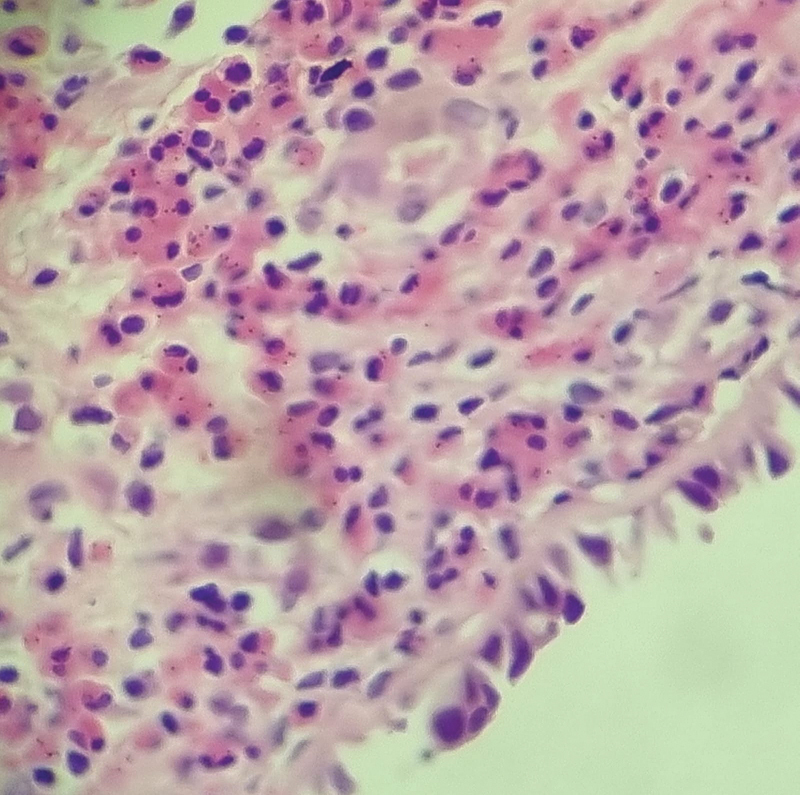
Demonstration of eosinophilic inflammation in the nasal polyp tissue; demonstration of more than 10 eosinophils per high-powered field 400x in a subject from the study.

Eosinophilic inflammation was demonstrated by the eosinophil count per high-powered field 400x ( Figure 3 ). Values >10 classify rhinosinusitis as eosinophilic, 1 and were found in 100% of the cases included herein. The values found ranged from 31 to 256. There was no significant association between eosinophil concentrations in polypoid tissue and postoperative SNOT-22 and UPSIT results. Patients with tissue eosinophils >50 had a lower preoperative UPSIT score.

## Discussion

There are two broad types of immune response in the human body: innate and adaptive. The pathophysiology of eCRS begins with the recruitment of eosinophils by a pathogen, eliciting an innate immune response with expression of interleukin (IL)-13 and thymic stromal lymphopoietin (TSLP) and a type 2 adaptive immune response which involves cytokines such as IL- 4, IL-5, and IL-13; these are precursors of type B lymphocytes, the cells that will produce IgE. 18 Synthetic corticosteroids act by inhibiting the synthesis and release of type B lymphocytes and the synthesis of TNF-alpha, interleukins 1 through 8, and interferon γ, thus inhibiting cytokine-mediated activation of T cells, which would differentiate into the Th2 (type 2) phenotype. Furthermore, corticosteroids disrupt the arachidonic acid cascade by inducing lipokines that inhibit phospholipase A2 and cyclooxygenases (COXs), thus preventing the synthesis of prostaglandins and prostacyclins. 19 All patients included in the present study had eCRS, a type of inflammation in which the inflammatory cascade is interrupted by corticosteroid therapy. In this sense, after wide opening of the paranasal sinuses, there was a significant improvement in quality of life and olfaction, as measured by SNOT-22 and UPSIT respectively. We believe the treating physician has an important role in not only performing wide opening of all paranasal sinuses, but also providing accurate guidance to patients about their condition and, above all, on the importance of continuing topical corticosteroid therapy in the postoperative period.

In a previous study carried out in Brazil, 20 a score below 10 points on the SNOT-22 was considered normal. Comparison of preoperative versus 1-year postoperative SNOT-22 scores in our sample show that no patient was classified within the normal range in the preoperative period, but at 1-year follow-up, 14 patients had a score <10 points, which would be considered healthy; 2 had a score of 11, which is near-normal; and all patients showed a noticeable improvement in quality of life, with an objective decrease in their scores. The preoperative UPSIT, as validated in Brazil, 21 showed that 15 patients had anosmia. One year after surgery, 6 patients were classified as having normosmia, 6 with mild microsmia, 4 with moderate microsmia, 2 with severe microsmia, and only 2 with anosmia. Notably, of these 2 patients who had anosmia at 1-year postoperative follow-up, one had a preoperative SNOT-22 score of 49 and the other had a preoperative score of 52; both had their scores reduced to 7 points at 1-year follow-up, which denotes a quality of life similar to that of healthy individuals. This demonstrates that, even though they did not have any objective improvement in olfaction according to the UPSIT, they experienced significant improvement in quality of life and reported a subjective improvement in their sense of smell as compared to the preoperative period.

The tissue eosinophil threshold has been discussed in the literature as a potential predictor of postoperative outcomes, but there is no consensus. Tao et al. 22 showed that a tissue eosinophil counts greater than 48 may be an independent risk factor for difficult-to-control eCRS at 1 year after surgery. Another study 23 suggested that an eosinophil count >55 could predict recurrence of CRS, and that patients with a tissue eosinophilia count >20 would need higher doses of steroids in nasal irrigation to control CRS at least during the first 6 months after surgery. The association of eCRS and asthma has also been implicated in worse surgical outcomes. 24 In our study, there was no worse prognosis for postoperative UPSIT and SNOT22 in patients with tissue eosinophils >50, nor in patients with asthma (p > 0.05). Patients with tissue eosinophils >50 had a lower preoperative UPSIT score, i.e., a high tissue concentration of eosinophils was associated with worse olfaction preoperatively. We demonstrated that appropriate surgery, when followed by adherence to postoperative high-volume nasal irrigation with budesonide, was able to effect improvements in olfaction and quality of life that persisted 1 year after surgery, regardless of tissue eosinophil counts and presence of comorbid asthma. Even intra-individual eosinophil counts can be questionable, since there is great variation in this parameter depending on where the polyp biopsy was collected.

Our study shows that clinical control of CRS can be achieved through a combination of surgery and nasal corticosteroid irrigation. In light of this finding, we believe that surgery still plays an important role in the approach to patients with eCRS, especially when patients receive appropriate postoperative education and adhere to postoperative treatment. Controlled studies with a larger number of patients and different CRS subtypes are needed to confirm these findings.

## Conclusion

In the present study, surgery with wide opening of the paranasal sinuses effectively controlled eCRS in patients who adhered to high-volume budesonide nasal irrigation postoperatively. There were significant improvements in quality of life and olfaction, which persisted at least up to one year postoperatively.
